# Lutein Attenuates Lipid Accumulation in Association with TFEB Nuclear Translocation and Autophagy–Lysosomal Responses in a Cellular Model of Hepatic Steatosis

**DOI:** 10.3390/antiox15081042

**Published:** 2026-08-21

**Authors:** Faride Saud, Daniel Cabrera, Catalina Valladares, Marjorie De la Fuente López, Rodrigo Maldonado-Agurto, Diego Irribarra-Tapia, Elisa Balboa

**Affiliations:** 1 Laboratory for Biomedical Research—LIBMED, Instituto de Investigación, Desarrollo e Innovación en Salud, Facultad de Medicina y Salud, Universidad Finis Terrae, Santiago 7501015, Chile; fsaud@uc.cl (F.S.);; 2 Facultad de Medicina, Centro de Investigación e Innovación Biomédica (CiiB), Universidad de los Andes, Monseñor Álvaro del Portillo 12455, Santiago 7620001, Chile; dacabrera@uandes.cl; 3 Fundación Arturo López Pérez OECI Cancer Center, Translational Medicine Laboratory, Centro de Investigación e Innovación en Cáncer, Santiago 7500921, Chile

**Keywords:** antioxidant, carotenoid, lutein, lipophagy, lysosomal exocytosis, TFEB, steatosis, lipid droplet

## Abstract

Lutein is a dietary xanthophyll carotenoid valued for its antioxidant properties that has been shown to benefit liver health and reduce hepatic lipid accumulation; however, this lipid-lowering effect cannot be fully attributed to its antioxidant activity, and the underlying mechanism remains poorly understood. Metabolic dysfunction-associated steatotic liver disease (MASLD) is characterized by excessive hepatic lipid accumulation, oxidative stress, and limited therapeutic options. Transcription factor EB (TFEB) coordinates the autophagy–lysosomal pathways involved in cellular lipid clearance, including lipophagy and lysosomal exocytosis, and is sensitive to the cellular redox state; however, whether the antioxidant lutein modulates TFEB-regulated lipid homeostasis remains unclear. HepG2 cells were exposed to free fatty acids (FFAs) to induce intracellular lipid accumulation and co-treated with lutein. TFEB localization and the expression of TFEB-related genes were assessed by immunofluorescence and qPCR, respectively. Immunofluorescence was also used to evaluate lipid droplet accumulation, LC3 content, and LAMP1 localization. Lipid droplet ultrastructure was analyzed by transmission electron microscopy, and extracellular triglyceride levels were measured as a functional readout of lipid extrusion. Lutein attenuated lipid droplet accumulation in FFA-treated cells, increased nuclear TFEB immunoreactivity, upregulated LC3 mRNA expression, and enhanced LC3 colocalization with lipid droplets. Chloroquine abolished the lipid-lowering effect of lutein, supporting an autophagy-dependent mechanism. In addition, lutein increased the abundance of LAMP1-positive compartments, while ultrastructural analysis and elevated extracellular triglyceride levels suggested enhanced lipid extrusion. These findings position the antioxidant lutein as a candidate natural compound whose lipid-lowering action is associated with TFEB nuclear translocation/activation and with the autophagy–lysosomal pathway, warranting further investigation in MASLD.

## 1. Introduction

Metabolic dysfunction-associated steatotic liver disease (MASLD), previously termed nonalcoholic fatty liver disease (NAFLD), has emerged as the most common cause of liver disease globally, affecting between 25 and 30% of the general population [1]. Prevalence rates are even higher in specific subpopulations with comorbidities such as obesity (90%) and patients with type 2 diabetes (76%) [2]. Moreover, the development of MASLD is often accompanied by features of metabolic syndrome, such as elevated plasma triglycerides (TGs), insulin resistance, hypertension, and decreased high-density lipoprotein (HDL) cholesterol.

The hallmark of MASLD is hepatic steatosis [3]. Hepatic steatosis is defined as the accumulation of triglycerides (TG), or neutral fats, in the cytoplasm of hepatocytes, accounting for at least 5% of the total liver weight. This TG accumulation results from an imbalance between lipid input and output, which are regulated through four major pathways: (i) uptake of circulating lipids, (ii) de novo lipogenesis (DNL), (iii) fatty acid oxidation (FAO), and (iv) export of lipids in very-low-density lipoproteins (VLDL) [4]. The dysregulation and contribution of each pathway to the pathogenesis of the disease are well documented. However, lipophagy, defined as the autophagic clearance of lipid droplets (LDs), has recently emerged as an important pathway contributing to hepatic lipid turnover and intracellular lipid homeostasis. Lipophagy is a key process responsible for lipid catabolism in the liver [5], regulating intracellular lipid levels in the hepatocyte. Lipophagy is a lysosome-mediated/dependent pathway for the breakdown of lipids/lipid droplets that complements the actions of cytosolic neutral lipases. Lipophagy is a relatively recently described pathway compared to the four described above. However, disturbances in lipophagy have been increasingly associated with MASLD and hepatic TG accumulation [6]. At the molecular level, lipophagy is regulated by a transcription-dependent mechanism in which the transcription factor EB (TFEB), a member of the basic helix-loop-helix leucine-zipper family of transcription factors, controls lysosomal biogenesis and autophagy by positively regulating genes belonging to the Coordinated Lysosomal Expression and Regulation (CLEAR) network [7].

Under nutrient-rich conditions, TFEB is isolated outside the nucleus in an inactive state by phosphorylation and accumulates in the cytoplasm. TFEB can be phosphorylated by mTORC1, AKT, GSK3β, ERK2, and MAPK4 [7]. Calcineurin can regulate the dephosphorylation of TFEB, which can be activated by lysosomal Ca^2+^ [8]. ER stress and reactive oxygen species (ROS) can promote the effects of calcineurin on TFEB directly or indirectly through the lysosomal Ca^2+^ channel mucolipin 1 (MCOLN1) [9].

Furthermore, TFEB exerts a central role in hepatic lipid metabolism by orchestrating a transcriptional program in response to fasting via the PGC1α-PPARα-lipophagy axis that mediates lipid catabolism. Moreover, evidence shows that the cAMP response element-binding protein (CREB) promotes lipophagy in the fasted state via direct transcriptional activation of TFEB and autophagy genes [10].

Previously it has been shown that TFEB overexpression and/or activation induce an increased number of autophagosomes and autophagic flux, as well as the generation of new lysosomes, leading to clearance of storage material in lysosomal storage disorders (LSDs) [11,12]. In addition, TFEB expression in vivo can be modulated via pharmacological induction [11,12,13,14]. Pharmacological activation of TFEB has been shown to reduce hepatic lipid accumulation and enhance β-oxidation in diet-induced MASLD animal models [15,16]. These findings support the rationale for targeting TFEB activation as a therapeutic strategy to mitigate hepatic steatosis. Accordingly, identifying nutritional or pharmacological modulators of TFEB activity becomes highly relevant. In this context, carotenoids have attracted considerable interest due to their beneficial effects on liver health [17].

Dietary carotenoids accumulate predominantly in the liver, from where they are incorporated into lipoproteins and released into the circulation, enabling their distribution to peripheral tissues [17]. In humans, circulating carotenoid levels are commonly used as an indirect indicator of their systemic availability and may reflect their potential hepatic bioactivity. In this context, the accumulation of these antioxidants and their bioactive metabolites in the liver supports their potential as a therapeutic strategy for liver diseases. Consistently, individuals who consume the highest amounts of carotenoids have the lowest risk of developing MASLD [18], and circulating levels of specific carotenoids, such as lutein, are inversely associated with severity of MASLD [19]. Importantly, lutein is one of the most abundant carotenoids in human plasma, together with lycopene, β-carotene, β-cryptoxanthin, α-carotene, and zeaxanthin [20]. It is a non-provitamin A carotenoid that belongs to the xanthophyll family and is found in spinach, kale, eggs, corn, and green leafy vegetables [21].

Lutein supplementation in rats fed a high-fat diet (HFD), a model of MASLD, recovered liver function, reduced lipid accumulation, and normalized hepatic lipid metabolism and insulin signaling through regulation of SIRT1, suggesting that lutein supplementation plays a potential role in preventing hepatic steatosis and insulin resistance [22]. Moreover, lutein reduced lipid peroxidation and pro-inflammatory cytokine production in the liver of guinea pigs fed a high-cholesterol diet [23], supporting the role of lutein as a hepatoprotective molecule.

Although there is evidence supporting the beneficial effects of lutein on liver health, the mechanisms by which lutein reduces lipid accumulation in hepatocytes remain poorly understood. Taken together, these findings suggest that lutein may exert its effects through activation of TFEB, thereby promoting lipid degradation in hepatocytes via lipophagy.

## 2. Materials and Methods

### 2.1. Cell Culture

HepG2 cells (human hepatocellular carcinoma; species: Homo sapiens; liver origin) were obtained from AddexBio Technologies (San Diego, CA, USA) (catalog no. C0015002). Cells were maintained in Minimal Essential Medium (MEM) with Earle’s Balanced Salt Solution (EBSS) and L-glutamine (Cytiva (Marlborough, MA, USA)), supplemented with 10% fetal bovine serum (FBS). Cells were cultured at 37 °C in a humidified atmosphere with 5% CO_2_.

Cells were passaged at 80–90% confluence using 1× trypsin and maintained under standard conditions, with medium renewal every 2–3 days. Before sample collection, cultures were routinely examined by phase-contrast microscopy to verify comparable cell attachment, morphology, and cell density across all experimental conditions.

### 2.2. Induction of Steatosis and Treatments

Steatosis was induced using a mixture of free fatty acids (FFAs), consisting of oleic acid and palmitic acid (2:1 ratio), conjugated to 12.5% bovine serum albumin (BSA) in MEM without FBS. Briefly, fatty acids were dissolved and incubated at 37 °C for 15–30 min prior to use. Final concentrations were 125 µM palmitic acid and 250 µM oleic acid.

Lutein (10010811, Cayman Chemical (Ann Arbor, MI, USA)) was prepared as a 4 mM stock solution in DMSO and used at a final concentration of 10 µM. For treatments, lutein was mixed with BSA-containing medium and added simultaneously with FFA. Cells were incubated for 12 h or 24 h depending on the experiment. The final DMSO concentration did not exceed 0.25% (*v*/*v*) and was matched across all conditions, including vehicle-treated controls.

Where indicated, cells were co-treated with rapamycin (10 nM) or chloroquine (10 µM) for 12 h. Control cells received vehicle treatment.

### 2.3. Immunofluorescence and Lipid Droplet Staining

Cells were seeded on poly-L-lysine-coated 12 mm coverslips and allowed to adhere for 24 h. After treatment, cells were washed three times with PBS and fixed with 4% formaldehyde for 15 min.

Lipid droplets were stained using BODIPY 493/503 0.01 mg/mL (Invitrogen (Thermo Fisher Scientific, Waltham, MA, USA) cat. no. D3922) for 30 min at 37 °C. Cells were then incubated in blocking/permeabilization buffer containing 1% BSA, 0.1% saponin, and 0.5% glycine.

Primary antibodies anti-TFEB 1:250 (Bethyl Laboratories (Montgomery, TX, USA), A303-672A), anti-LAMP1 1:50 (Abcam (Cambridge, UK), ab-25630), anti-LC3 1:500 (Cell Signaling Technology (Danvers, MA, USA), 12741), anti-PLIN2 1:500 (Abcam, ab-108323) were incubated overnight at 4 °C. After washing, cells were incubated with Alexa Fluor-conjugated secondary antibodies (1:500) for 1 h at room temperature.

Nuclei were stained with Hoechst 1:10,000 (Sigma-Aldrich (St. Louis, MO, USA), H6024) for 5 min. Coverslips were mounted using Fluoromount-G (SouthernBiotech, (Birmingham, AL, USA) 0100-01).

### 2.4. Confocal Microscopy and Image Analysis

Images 1 and 6 were acquired using a Nikon C2+ confocal microscope (Nikon, Tokyo, Japan) with a 40× objective. High-resolution imaging and 3D reconstruction (Figure 3) were performed using Airyscan (ZEISS, Oberkochen, Germany).

Image analysis was conducted using ImageJ/Fiji software (ImageJ version 1.54p). Lipid accumulation was quantified by measuring BODIPY or Nile Red fluorescence intensity. TFEB nuclear translocation was assessed by calculating the nuclear-to-cytoplasmic fluorescence ratio.

Colocalization between LC3 and lipid droplets was analyzed using Pearson’s correlation coefficient, calculated in ImageJ/Fiji on background-subtracted, thresholded images using individual cells as regions of interest. Representative images were obtained from three independent experiments. LAMP1 redistribution was quantified as the percentage of signal localized in the perinuclear region relative to total cellular fluorescence.

### 2.5. RNA Extraction and Quantitative PCR (qPCR)

Total RNA was extracted using TRIzol reagent (Invitrogen, Thermo Fisher Scientific, (Waltham, MA, USA) cat. no. 15596018) according to the manufacturer’s instructions. cDNA synthesis was performed from equal amounts of RNA.

Quantitative PCR was carried out using gene-specific primers for *TFEB*, *LAMP1*, *SQSTM1*, *MAP1LC3B*, *ATG9A*, and *BECN1* (Supplementary Table S1). Gene expression was normalized to the *GAPDH* housekeeping gene, whose primer sequences are listed in Supplementary Table S1. Data were analyzed using the REST method [24].

### 2.6. Transmission Electron Microscopy (TEM)

Cells were fixed and processed according to standard protocols for transmission electron microscopy. Ultrathin sections were analyzed to evaluate cellular ultrastructure.

Lipid droplet number and morphology were quantified from representative images. The distance between lipid droplets and the plasma membrane (LD-to-PM distance) was measured, and lipid droplets located near the plasma membrane (<0.5 um) were defined as submembranous LD.

### 2.7. Triglyceride Quantification

Culture medium triglycerides were measured using a colorimetric triglyceride assay kit (MAK266-1KT, Sigma-Aldrich) according to the manufacturer’s instructions. Briefly, triglycerides were hydrolyzed by lipase to free fatty acids and glycerol, and the released glycerol was oxidized to generate a colorimetric product measured at 570 nm in a microplate reader. Standard curves were generated using serial dilutions of the triglyceride standard included in the kit. Sample triglyceride concentrations were calculated from the standard curve, normalized to the volume of medium, and expressed as nmol/mL. All assays were performed in technical duplicates.

### 2.8. Western Blot Analysis

After treatment, cells were washed twice with ice-cold PBS and lysed in RIPA buffer supplemented with protease and phosphatase inhibitors. Lysates were cleared by centrifugation (12,000× *g*, 10 min, 4 °C) and protein concentration was determined with a BCA protein assay. Equal amounts of protein were resolved on 15% SDS-PAGE gels, which allow the separation of LC3-I from LC3-II, and transferred onto PVDF membranes. Membranes were blocked with 5% non-fat dry milk in TBS–Tween 0.1% and incubated overnight at 4 °C with primary antibodies against LC3 (Cell Signaling, 12741), SQSTM1/p62 (Invitrogen, MA5-27800) and GAPDH (Novus Biologicals, Centennial, CO, USA, cat. NB100-56875), used as a loading control, followed by HRP-conjugated secondary antibodies for 1 h at room temperature. Immunoreactive bands were revealed by enhanced chemiluminescence. Autophagic flux was assessed by comparing LC3-II and p62 levels in the absence and in the presence of chloroquine (10 µM, 12 h), which blocks lysosomal degradation; rapamycin (10 nM, 12 h) was included as a positive control for autophagy induction.

### 2.9. siRNA-Mediated TFEB Silencing

HepG2 cells were seeded in 12-well plates and transfected at 60–70% confluence with a non-targeting control siRNA (siRNA-nt) or with one of three independent siRNA sequences targeting human TFEB (TFEB Human siRNA Oligo Duplex (Locus ID 7942, OriGene Technologies (Rockville, MD, USA))). using siTran 2.0 siRNA transfection reagent (TT320001, Origene), according to the manufacturer’s instructions. Silencing efficiency was determined 48 h post-transfection by qPCR (Supplementary Figure S4A). Forty-eight hours after transfection, cells were treated for 24 h with vehicle, lutein (10 µM), FFAs, or FFAs plus lutein, after which triglycerides and β-hexosaminidase activity were measured in the culture medium.

### 2.10. β-Hexosaminidase Release Assay

Lysosomal exocytosis was estimated by measuring the activity of the lysosomal enzyme β-hexosaminidase released into the culture medium relative to total cellular activity. After treatment, the culture medium was collected and cleared by centrifugation, and the corresponding cell monolayers were lysed in PBS containing 0.2% Triton X-100. Equal volumes of medium and lysate were incubated with p-nitrophenyl-N-acetyl-β-D-glucosaminide in citrate/phosphate buffer (pH 4.5) for 30 min at 37 °C. Reactions were stopped with glycine/carbonate buffer (pH 10.4) and absorbance was read at 405 nm in a microplate reader. Results are expressed as the percentage of released (extracellular) activity relative to total (extracellular plus intracellular) activity.

### 2.11. Statistical Analysis

Data are presented as mean ± SEM from at least three independent biological experiments. For all immunofluorescence- and transmission electron microscopy (TEM)-based quantifications, the independent biological experiment, rather than the individual cell, cellular structure, or image, was considered the statistical unit (n = 3). Multiple cells, cellular structures, and images acquired within each experiment were used only as technical observations and were averaged before statistical analysis.

Comparisons between two groups were performed with Student’s *t*-test and comparisons among three or more groups with one-way ANOVA followed by Tukey’s post-hoc test; qPCR data were analysed with the pairwise fixed reallocation randomisation test implemented in REST. Analyses were performed in GraphPad Prism. A *p*-value < 0.05 was considered statistically significant.

## 3. Results

### 3.1. Lutein Induces TFEB Nuclear Translocation and Activates TFEB Transcriptional Program

**Figure 1:**
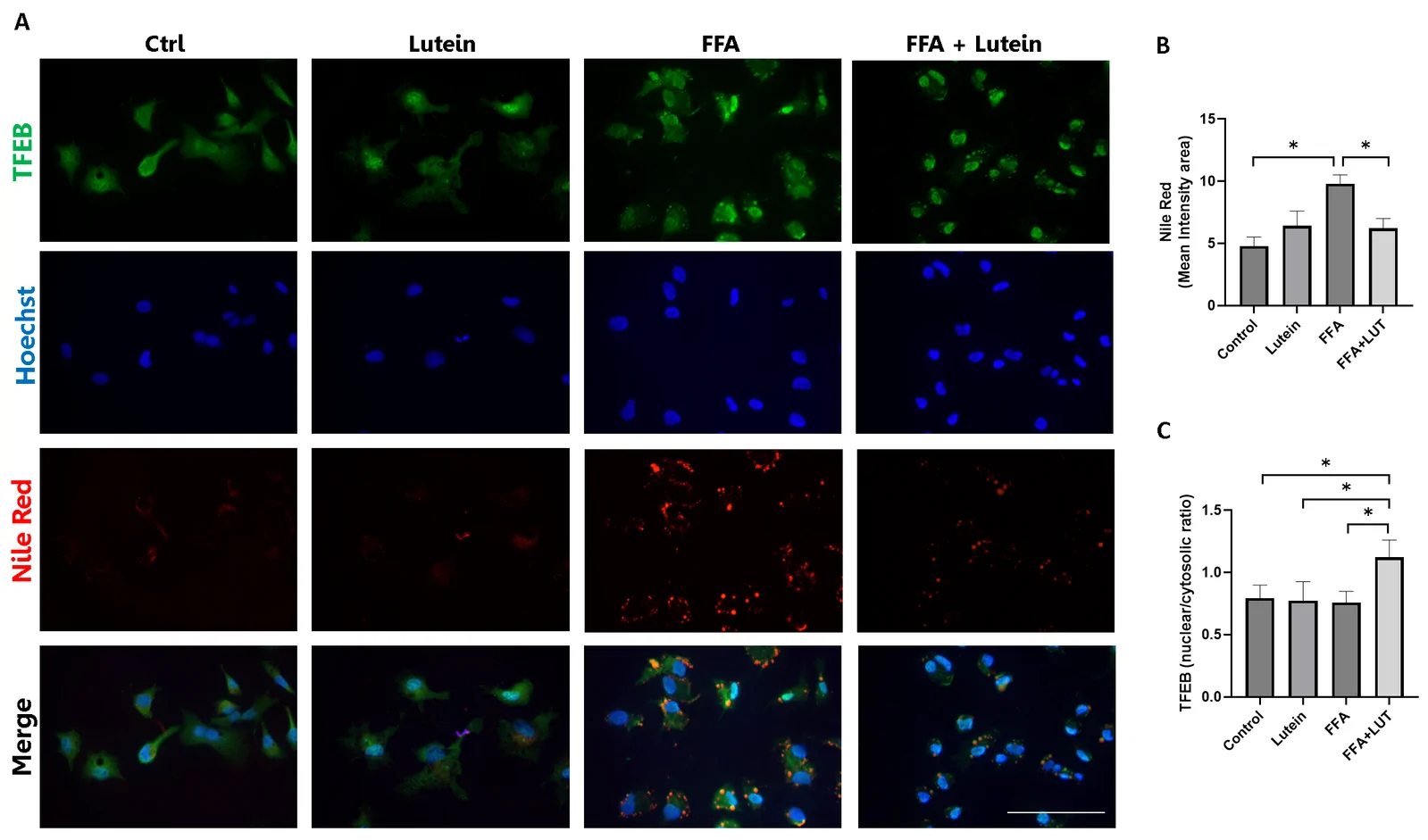
Effect of free fatty acids and lutein on TFEB localization in an in vitro model of hepatic steatosis. HepG2 cells were treated for 24 h with a mixture of free fatty acids (FFAs; palmitic acid 125 µM and oleic acid 250 µM) and/or lutein (10 µM). (**A**) Representative immunofluorescence images showing TFEB (green), nuclei stained with Hoechst (blue), and lipid droplets stained with Nile Red (red). Scale bar is 100 μm. (**B**) Quantification of Nile Red fluorescence intensity. (**C**) Quantification of the nuclear-to-cytoplasmic TFEB fluorescence intensity ratio. Data are presented as mean ± SEM of n = 3 independent experiments; four images were analysed per condition in each experiment and averaged. * *p* < 0.05.

To establish an in vitro model of MASLD and assess whether lutein modulates the nuclear localization of TFEB, HepG2 cells were treated with free fatty acids (FFAs), inducing intracellular lipid accumulation and the development of steatosis (Figure 1). In FFA-treated cells, co-treatment with lutein reduces Nile Red fluorescence intensity, indicating a decrease in lipid droplet (LD) accumulation in these cells (Figure 1B). TFEB expression and subcellular localization were evaluated by immunofluorescence (Figure 1A,C). We found that 24-h treatment with FFAs plus lutein increased nuclear TFEB immunostaining without altering total cellular TFEB immunostaining.

**Figure 2:**
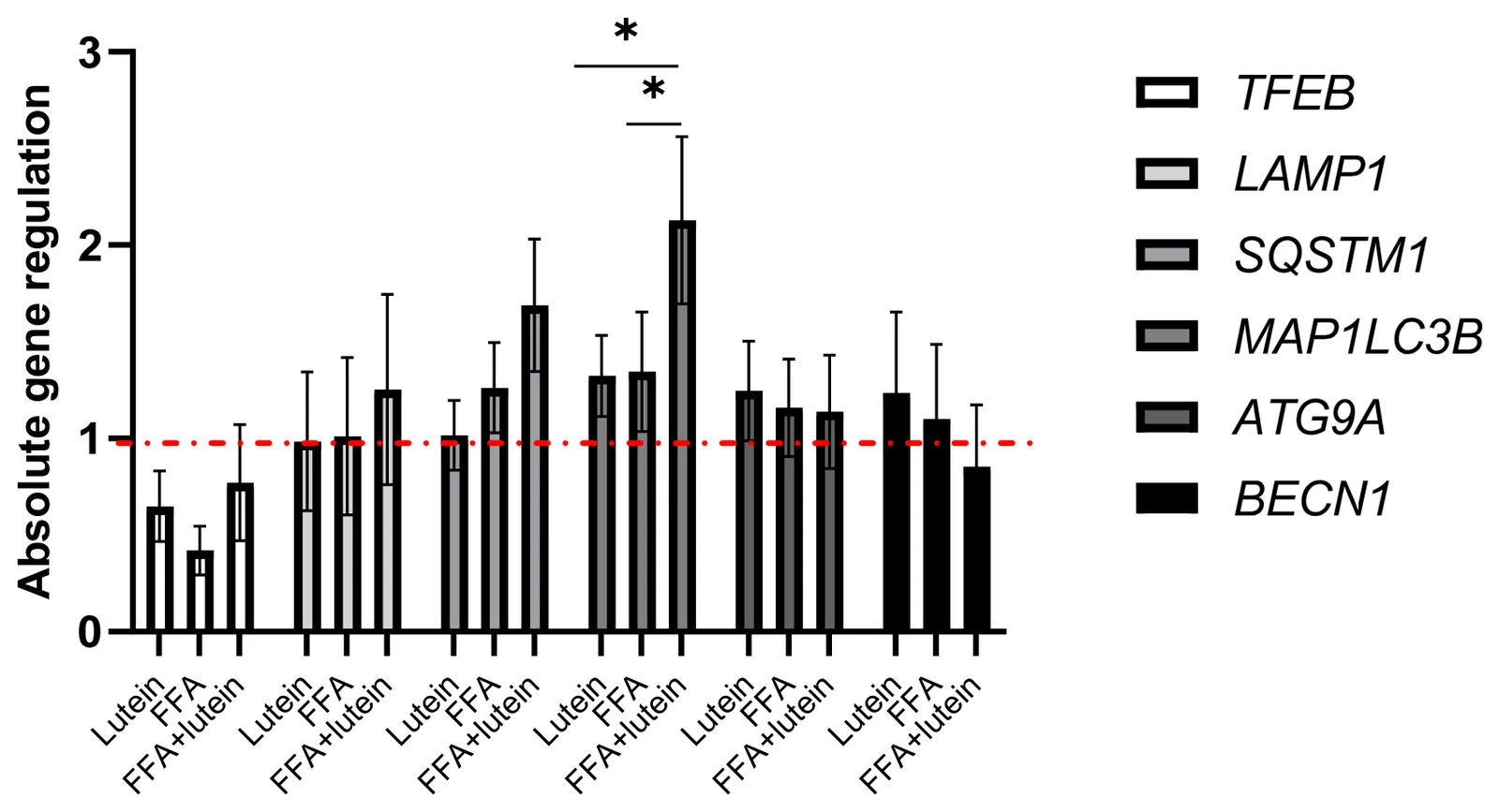
TFEB target gene expression in HepG2 cells. HepG2 cells were treated for 24 h with a mixture of free fatty acids (FFAs; palmitic acid 125 µM and oleic acid 250 µM) and/or lutein (10 µM). The mRNA expression levels of *TFEB* and of its target genes (*LAMP1*, *SQSTM1*/*p62*, *MAP1LC3B*/*LC3*, *ATG9A* and *BECN1*/*Beclin 1*) were analyzed by quantitative PCR (qPCR) using total RNA extracted from HepG2 cells. Gene expression was normalized to the *GAPDH* housekeeping gene (Supplementary Table S1). Relative gene expression (absolute regulation) was calculated using the Relative Expression Software Tool (REST, version 2), which applies a pairwise fixed reallocation randomization test to determine expression ratios between groups. Data are presented as mean ± SEM. A red dashed line indicates control expression levels, which were normalized to 1. * Indicates a statistically significant difference (*p* < 0.05).

To determine whether lutein-induced TFEB nuclear translocation impacts the expression of TFEB target genes, we assessed the mRNA levels of *TFEB*, *LAMP1*, *SQSTM1*/*p62*, *MAP1LC3B*/*LC3*, *ATG9A* and *BECN1*/*Beclin 1* by qPCR (Figure 2). We observed a slight increase in *LAMP1* and *SQSTM1*/*p62* expression in cells treated with FFAs and lutein compared to control and FFA-treated cells; however, these differences did not reach statistical significance. In contrast, lutein treatment significantly increased *MAP1LC3B*/*LC3* gene expression in FFA-treated cells. No changes were detected in *TFEB*, *ATG9A* or *BECN1* expression.

Together, these results suggest that lutein promotes TFEB activation and selectively enhances the expression of specific TFEB target genes.

### 3.2. Lutein Promotes LC3 Redistribution and Its Association with Lipid Droplets in FFA-Treated Cells

**Figure 3:**
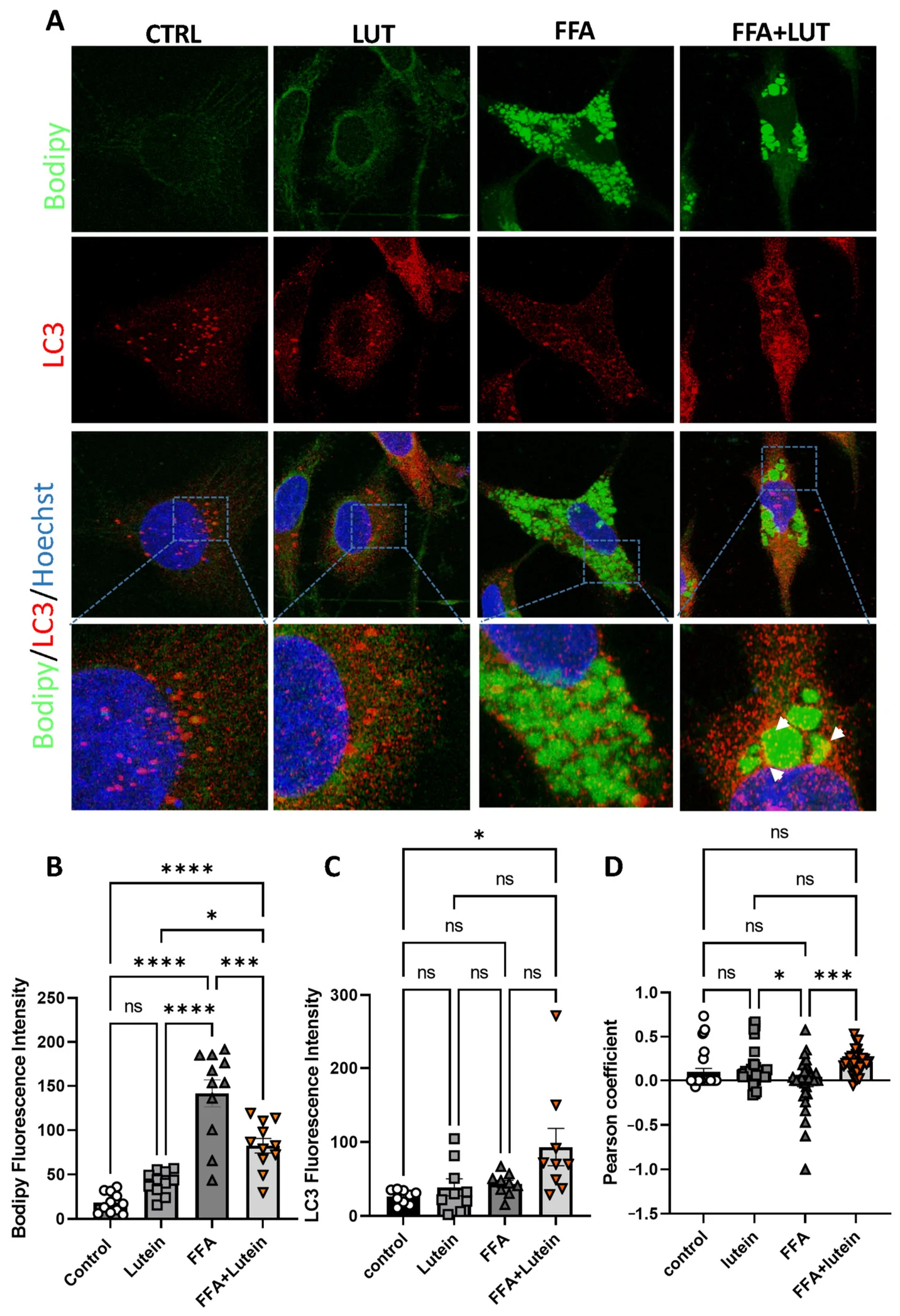
Lutein modulates lipid accumulation and LC3 distribution in an in vitro model of steatosis. HepG2 cells were treated for 12 h with free fatty acids (FFAs) to induce steatosis and/or with lutein (10 µM). (**A**) Representative immunofluorescence images acquired by confocal microscopy with 3D reconstruction using Airyscan, showing LC3 (red), lipid droplets stained with BODIPY (green), and nuclei stained with Hoechst (blue). White arrows indicate LC3 colocalization with lipid droplets. (**B**) Quantification of BODIPY fluorescence intensity. (**C**) Quantification of LC3 fluorescence intensity. (**D**) Colocalization analysis between LC3 and BODIPY, expressed as Pearson’s correlation coefficient, performed using ImageJ. Data are presented as mean ± SEM of n = 3 independent experiments; all cells and fields analysed within one experiment were averaged, so that the independent experiment, and not the individual cell or image, is the statistical unit. Statistical significance is indicated as follows: **** *p* < 0.0001; *** *p* < 0.001; * *p* < 0.05. ns, not significant.

Consistent with the observed increase in LC3 mRNA levels in FFA- and lutein-treated cells, we next evaluated LC3 protein distribution by immunofluorescence (Figure 3). To determine whether the previously observed reduction in LD is associated with increased LC3 and potentially enhanced lipophagy, we assessed the colocalization of LC3 with LD stained with BODIPY. This analysis was performed at 12 h post-treatment to capture LD within autophagic structures, as LD content had already been reduced at 24 h in lutein-treated cells.

We observed a reduction in LD content in cells treated with FFAs and lutein compared to FFAs alone (Figure 3A,B). In addition, LD displayed a more organized and clustered distribution in the perinuclear region in lutein-treated cells. Importantly, colocalization analysis revealed a significant increase in LC3–LD overlap in cells treated with FFAs and lutein compared to those treated with FFAs alone (Figure 3A,D), indicating an increased association of lipid droplets with the autophagic machinery.

Because LC3 mRNA levels and LC3–LD colocalization cannot by themselves discriminate between increased autophagosome formation and impaired autophagosome clearance, we performed a Western blot analysis of LC3-I/LC3-II and p62 (SQSTM1) in Control, lutein, FFAs, and FFAs plus lutein conditions, in the absence or presence of chloroquine (CQ, 10 µM) or rapamycin (Rap, 10 nM) (Supplementary Figure S3). FFAs alone increased LC3-II without a concomitant decrease in p62, whereas co-treatment with lutein was associated with higher LC3-II levels and lower p62 abundance compared with FFAs alone. Consistent with this pattern, CQ treatment resulted in a greater accumulation of both LC3-II and p62 in FFAs plus lutein-treated cells than in cells treated with FFAs alone. Although these data should be considered preliminary (n = 1), the observed pattern, together with the increased colocalization of LC3 with lipid droplets, is compatible with the involvement of autophagy in the observed phenotype.

### 3.3. The Lipid-Lowering Effect of Lutein Is Prevented by Chloroquine in FFA-Treated HepG2 Cells

To determine whether the effects of lutein are associated with autophagic activity, additional treatments were performed using rapamycin and chloroquine. Ultrastructural analysis by transmission electron microscopy confirmed the observations obtained.

**Figure 4:**
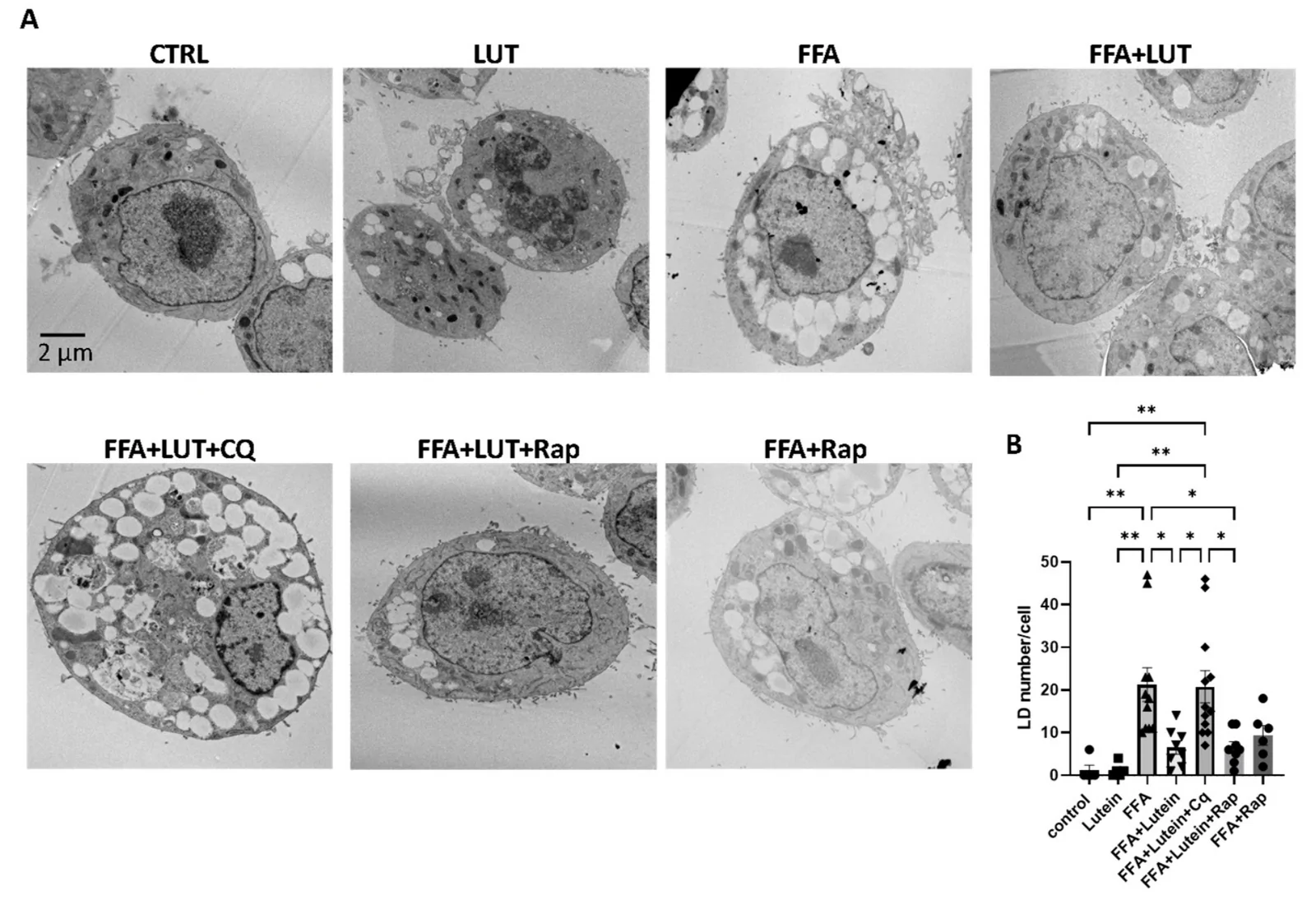
Lutein modulates lipid droplet reduction in HepG2 cells treated with free fatty acids. (**A**) Representative transmission electron microscopy images of HepG2 cells treated for 24 h with free fatty acids (FFAs; oleic acid and palmitic acid, 2:1), in the absence or presence of lutein (10 µM). Where indicated, cells were co-treated with chloroquine or rapamycin for 12 h. (**B**) Quantification of lipid droplet (LD) number from three representative transmission electron microscopy images. Data are presented as mean ± SEM (n = 3). * Indicates a statistically significant difference (* *p* < 0.05, ** *p* < 0.01).

Lutein-treated cells exhibited a clear reduction in the number of lipid droplets (LD), along with a more organized intracellular distribution (Figure 4). Notably, cells treated with rapamycin displayed a similar morphological phenotype to those treated with lutein. In contrast, co-treatment with chloroquine abrogated the effects of lutein, resulting in a marked accumulation of LD comparable to that observed in FFA-treated cells.

Taken together, these findings suggest that lutein modulates lipid homeostasis in HepG2 cells through an autophagy-dependent mechanism, as inhibition of autophagic flux with chloroquine prevents the lipid-lowering effect of lutein.

### 3.4. Lutein Enhances Lipid Droplet Proximity to the Plasma Membrane and Increases Extracellular Lipid Release

**Figure 5:**
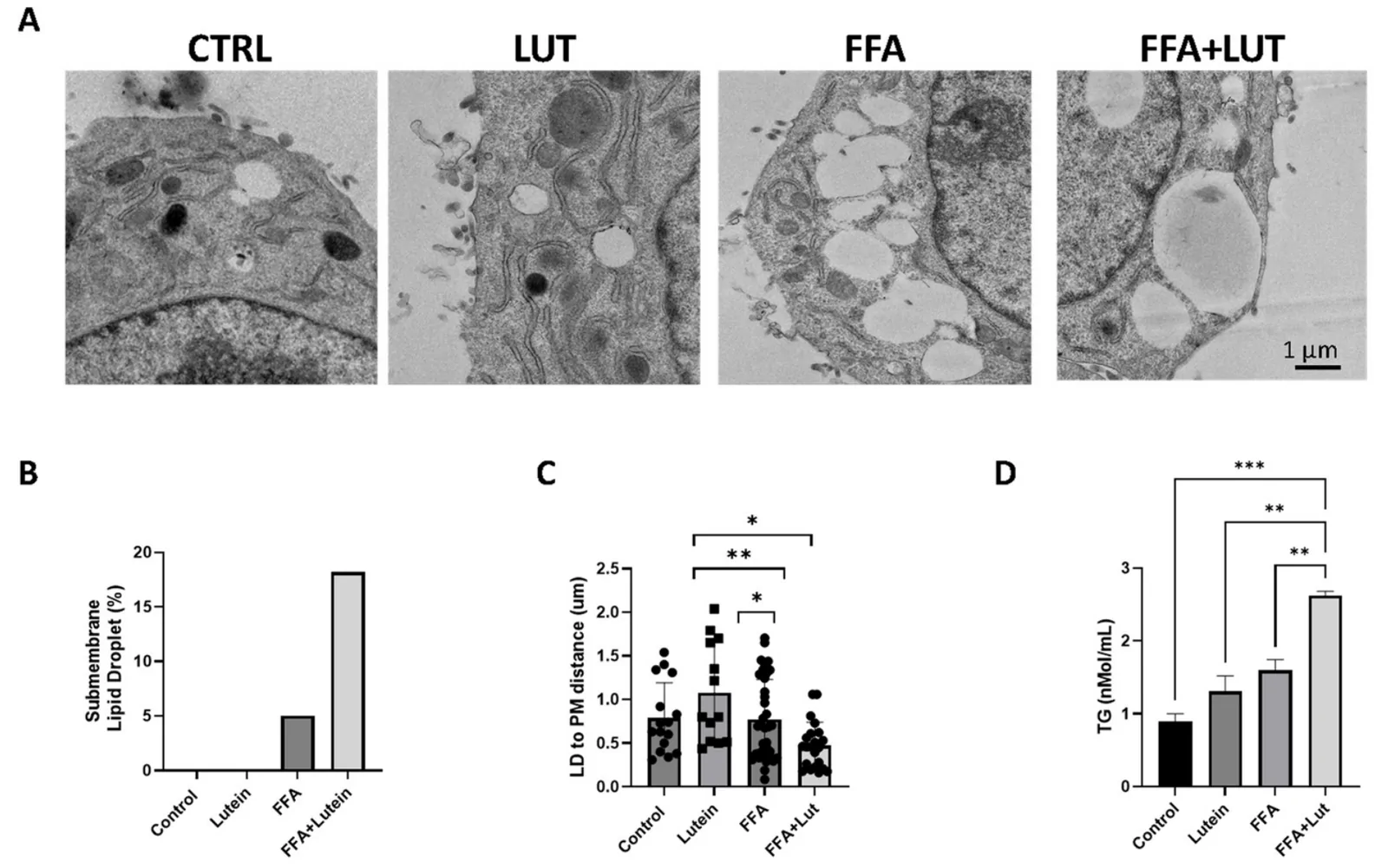
Lutein increases lipid droplet proximity to the plasma membrane and extracellular triglyceride release. (**A**) Representative transmission electron microscopy images of HepG2 cells treated for 24 h with free fatty acids (FFAs; oleic acid/palmitic acid, 2:1), in the absence or presence of lutein (10 µM). (**B**) Quantification of lipid droplets (LDs) located near the plasma membrane, expressed as the percentage of total LDs with a plasma membrane distance <0.5 µm. (**C**) Distance between LDs and the plasma membrane. (**D**) Triglyceride (TG) levels in culture medium, expressed as nmol/mL. Data are presented as mean ± SEM (n = 3). * Indicates a statistically significant difference (* *p* < 0.05, ** *p* < 0.01, *** *p* < 0.001).

To investigate a potential alternative mechanism of lipid droplet clearance, we next evaluated the lutein effects in extracellular lipid release. To address this, we performed ultrastructural analyses by transmission electron microscopy as a morphological-functional approach. Considering that the likelihood of a lipid droplet (LD) being released via exocytosis increases as it approaches the plasma membrane, we quantified the number of LDs located in close proximity to the plasma membrane, defined as submembranous LDs (Figure 5B), as well as the distance between LD and the plasma membrane (LD-to-PM distance, Figure 5C). We observed that in cells treated with FFAs and lutein, the number of submembranous LDs was increased compared with control and FFA-treated cells, and the LD-to-PM distance was significantly reduced compared with FFA-treated cells alone, suggesting a spatial redistribution of LDs towards the cell periphery.

To complement these observations, we quantified triglyceride (TG) levels in the culture medium as a functional readout of extracellular lipid release. In agreement with the ultrastructural findings, lutein treatment significantly increased TG levels in the medium of FFA-treated cells compared to cells not treated with lutein (Figure 5D).

To further investigate whether extracellular lipid release and lysosomal exocytosis might be associated with the observed phenotype, and to explore the potential involvement of TFEB, we performed two additional exploratory experiments (Supplementary Figure S4). In cells transfected with control (siRNA-nt) or TFEB siRNA we measured TG in the culture medium and the release of β-hexosaminidase into the medium as an indirect marker of lysosomal exocytosis. In control-transfected cells, lutein increased extracellular TG, reproducing the effect shown in Figure 5D, and this increase was abolished by TFEB silencing (Supplementary Figure S4B). Extracellular β-hexosaminidase activity was likewise increased by lutein alone, and this increase was also lost upon TFEB silencing (Supplementary Figure S4C). Unexpectedly, no clear change in β-hexosaminidase release was detected in the FFAs or FFAs plus lutein conditions. Given that these experiments were performed with a single biological replicate and partial TFEB knockdown, these findings should be considered exploratory and interpreted with caution.

Taken together, these exploratory findings are compatible with the possibility that lutein-associated lipid clearance may involve extracellular lipid release in addition to autophagy-related responses.

### 3.5. Lutein Alters LAMP1 Localization, Suggesting Potential Changes in Lysosomal Activity

**Figure 6:**
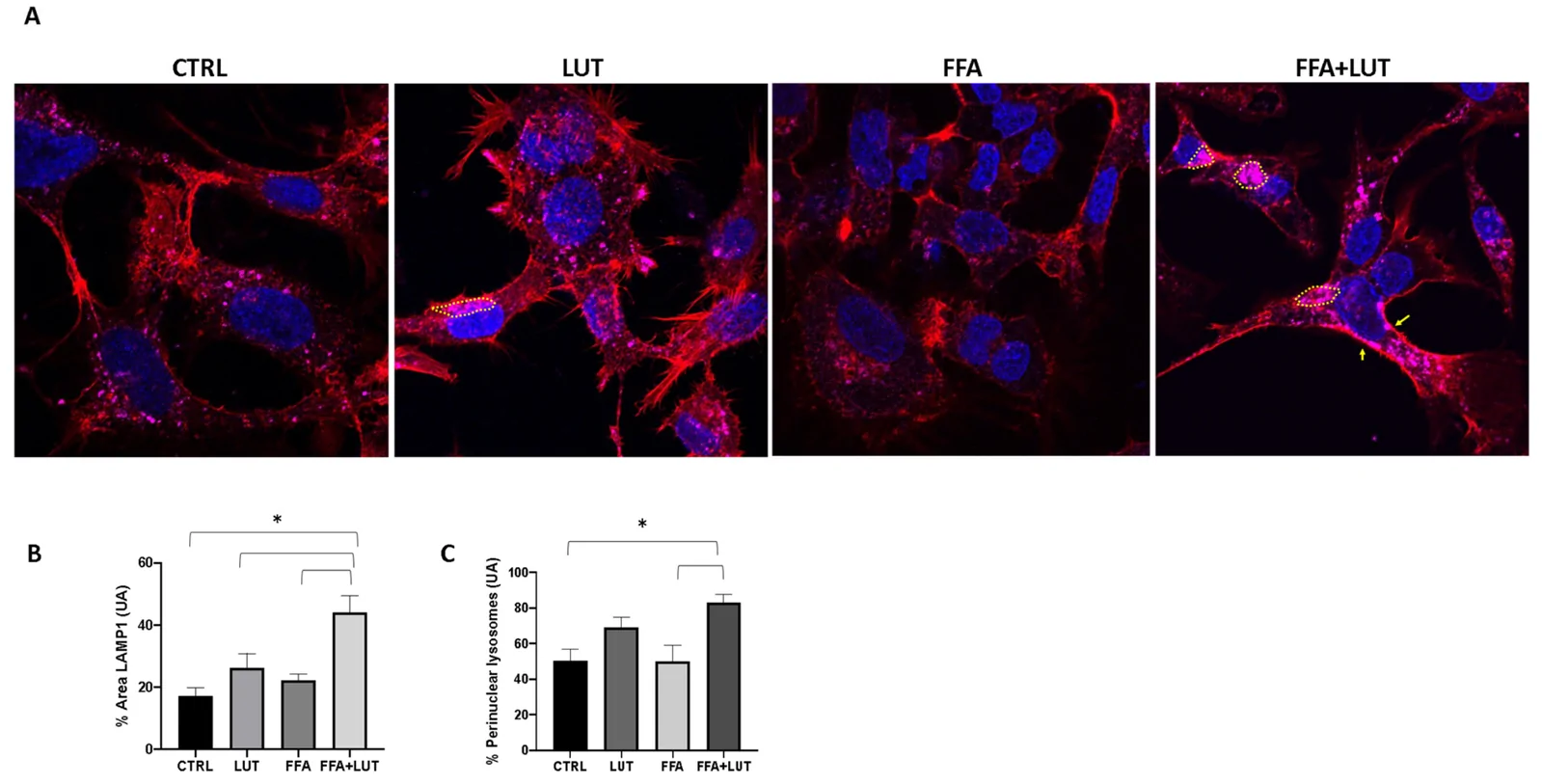
Lutein modulates the distribution and intensity of LAMP1 in FFA-treated HepG2 cells. (**A**) Representative immunofluorescence images of HepG2 cells treated with free fatty acids (FFAs; oleic acid and palmitic acid, 2:1) in the absence or presence of lutein (10 µM). LAMP1 was labeled in magenta, lipid droplets (LDs) were immunoassayed with perilipin 2 (green), the cytoskeleton was stained with phalloidin (red), and nuclei were counterstained with Hoechst (blue) Perinuclear lysosomal clustering is indicated by yellow dashed lines, while arrows indicate lysosomal staining close to the cell periphery. (**B**) LAMP1 fluorescence intensity was quantified using ImageJ, and data were plotted using GraphPad Prism 9. (**C**) Total cellular LAMP1 fluorescence intensity and perinuclear LAMP1 localization were quantified. Perinuclear localization is expressed as the percentage of total cellular LAMP1 fluorescence within the perinuclear region. Data are presented as mean ± SEM (n = 3). (* *p* < 0.05).

To further assess whether lutein influences lysosomal distribution, we evaluated the subcellular distribution and immunostaining pattern of LAMP1, a well-established lysosomal marker (Figure 6). Immunofluorescence analysis revealed that lutein treatment in FFA-treated HepG2 cells altered both the intensity and subcellular localization of the LAMP1 signal. Quantitative analysis showed increased LAMP1 fluorescence intensity in cells treated with lutein compared to control and FFAs alone (Figure 6 and Supplementary Figure S1), suggesting a potential enhancement of lysosomal content. In addition, lutein treatment induced a redistribution of LAMP1 signal, with a higher fraction of lysosomes localized in the perinuclear region (Figure 6C).

These observations suggest that lutein is associated with a reorganization of the lysosomal compartment.

## 4. Discussion

Metabolic dysfunction-associated steatotic liver disease (MASLD) is characterized by excessive hepatic lipid accumulation and limited therapeutic options beyond lifestyle changes. In this context, dietary antioxidants and isolated natural compounds have attracted increasing interest as potential adjuvant strategies, due to their pleiotropic effects on oxidative stress, inflammation, and cellular metabolism. In the present study, we found that lutein, a dietary carotenoid with antioxidant properties, modulates lipid homeostasis in an in vitro model of hepatocellular steatosis, in association with nuclear translocation of transcription factor EB (TFEB), autophagy-related responses, and morphological features compatible with altered lipid clearance.

We first found that lutein prevents lipid droplet accumulation during free fatty acid (FFA) treatment in HepG2 cells and promotes TFEB nuclear translocation without changing the overall cellular TFEB immunofluorescence signal. These results are in line with previous studies showing that TFEB activation reduces steatosis in hepatocytes [25,26].

TFEB is a redox-sensitive transcription factor; reactive oxygen species (ROS) can promote its dephosphorylation and nuclear translocation, directly or indirectly through calcineurin and the lysosomal Ca^2+^ channel MCOLN1 [8,9]. This raises the question of whether the TFEB activation we observed simply reflects an oxidative-stress response rather than a specific effect of lutein. To address this, we used a TFEB-GFP reporter system in which oxidative stress was induced with hydrogen peroxide (Supplementary Figure S2). As expected, H_2_O_2_ increased intracellular ROS, measured by DHE fluorescence, and was accompanied by partial TFEB nuclear translocation. Co-treatment with lutein markedly reduced the DHE signal, confirming its antioxidant activity in this system, yet TFEB accumulated more strongly in the nucleus. This dissociation is informative; lutein promotes TFEB nuclear localization even under conditions of lowered ROS, indicating that its effect on TFEB is not merely a downstream consequence of oxidative-stress signaling. Rather, lutein appears to engage TFEB through additional, ROS-independent inputs while simultaneously relieving the oxidative burden associated with lipid overload. This dual action, reducing oxidative stress while sustaining TFEB activation, may be particularly relevant in MASLD, where lipotoxicity and ROS accumulation impair autophagic-lysosomal function.

Based on increased nuclear TFEB immunostaining, we expected to observe increased mRNA levels of *TFEB* and its downstream target genes; however, at the 24 h time point evaluated here, this transcriptional response may not have been fully captured.

In our study, lutein selectively upregulated *LC3* mRNA, whereas *TFEB*, *LAMP1*, and *p62* showed only modest, non-significant changes. This apparent discrepancy may reflect the limited sample size and the resulting variability among experimental replicates, or it may indicate that the transcriptional response occurs at a different time window than the one analyzed. Notably, TFEB nuclear translocation was already evident at 24 h, suggesting that the signaling event had occurred, but that additional time may be required for robust induction of downstream transcripts. At the same time, the reduction in lipid droplet content observed at this point indicates that lutein had already initiated functional changes in lipid handling. Therefore, the absence of significant changes in some TFEB target genes should not be interpreted as evidence of pathway inactivity. In this regard, it is also important to note that although *LAMP1* mRNA was not significantly altered, LAMP1 immunofluorescence intensity was increased in lutein-treated cells (Figure 6), a finding that is consistent with modulation of lysosome-related responses despite the absence of detectable changes at the mRNA level.

Additional evidence compatible with the involvement of autophagy was obtained by immunoblot analysis of LC3 and p62 (Supplementary Figure S3). Although these experiments were performed only once (n = 1) and therefore should be interpreted cautiously, lutein increased LC3-I to LC3-II conversion while reducing p62 protein abundance, a pattern compatible with increased autophagy-related turnover. In contrast, FFA-treated cells accumulated p62, suggesting impaired autophagic degradation under steatotic conditions. These preliminary findings are consistent with the imaging data and support the interpretation that the effects of lutein are associated with autophagy-related responses. However, additional studies will be required to determine whether lutein primarily increases autophagosome formation, enhances autophagic flux, or affects both processes.

Consistent with these changes, we observed an increase in LC3-lipid droplet (LD) colocalization in FFA- and lutein-treated cells, indicative of enhanced lipophagy [27]. The marked reduction in LD content and the more organized distribution of droplets further support a role for autophagy-mediated lipid degradation [28]. Importantly, the lipid-lowering effect of lutein was abolished by chloroquine, a late-stage autophagy inhibitor, while the autophagy inducer rapamycin recapitulated the phenotype, suggesting that lutein promotes lipid clearance through an autophagy-dependent mechanism. These findings align with the growing body of evidence that compounds can modulate hepatic lipid metabolism via TFEB activation [13,29] and autophagy-related pathways, and they position lutein as a candidate phytopharmacological agent worthy of further investigation.

To further explore whether TFEB contributes functionally to lutein-induced lipid clearance, we performed preliminary TFEB knockdown experiments using siRNA (Supplementary Figure S4). Although these experiments were conducted with a single biological replicate and therefore cannot be considered conclusive, TFEB silencing abolished the increase in extracellular triglycerides induced by lutein in FFA-treated cells. While these findings require confirmation in independent experiments, together with the observed TFEB nuclear translocation, LC3-LD colocalization, and the loss of lutein’s lipid-lowering effect in the presence of chloroquine, they are consistent with the possibility that the lipid-exporting effect of lutein is associated, at least in part, with TFEB-associated autophagy–lysosomal responses.

Moreover, the increased extracellular triglyceride levels observed in lutein-treated cells suggest enhanced lipid export, supporting a role for lipid droplets beyond intracellular storage in the maintenance of cellular lipid homeostasis. This finding aligns with the notion that lysosomal-derived fatty acids are exported rather than directly trafficked to mitochondria for β-oxidation or ER/lipid droplets for re-esterification, as intracellular FAs are highly toxic and central to lipotoxicity [30]. Thus, lipid droplet efflux likely serves as a protective strategy to alleviate FA burden when metabolic capacity is overwhelmed, enabling cells to regulate FA reuptake based on demand, reduce intracellular toxicity, or redistribute energy to neighboring cells during high FA influx conditions like fasting. It remains unclear whether this lipid droplet efflux is beneficial in a more physiological context, where neighboring cells of hepatocytes, such as stellate cells, could become activated, thereby promoting liver inflammation.

To obtain a more direct functional readout associated with lysosomal exocytosis, we measured extracellular β-hexosaminidase activity (Supplementary Figure S4). Interestingly, lutein alone increased enzyme activity in the extracellular medium, supporting enhanced lysosomal exocytosis under basal conditions. Unexpectedly, this increase was not observed in FFA-treated cells supplemented with lutein, despite the elevated extracellular triglyceride levels. Therefore, our data do not provide evidence that lysosomal exocytosis accounts for the increased extracellular triglycerides observed under steatotic conditions. Instead, these findings suggest that additional mechanisms, including conventional lipid secretion, extracellular vesicles, or other pathways, cannot be excluded. Further studies will be required to clarify the origin of the extracellular triglycerides and the contribution of lysosomal exocytosis to this phenotype.

Furthermore, lutein treatment altered the distribution and intensity of the lysosomal marker LAMP1, with increased fluorescence intensity and a higher proportion of lysosomes localized in the perinuclear region of the cell. These changes are consistent with a potential remodeling of lysosomal organization and may reflect changes in lysosome-related responses, which may facilitate lipid droplet engulfment and/or lysosomal trafficking required for autophagy [31]. Taken together with TFEB nuclear localization, LC3-LD colocalization and the LAMP1 redistribution, data point to a broader reorganization of lysosomal-autophagic machinery that may underlie lutein’s effects on lipid clearance.

Although we demonstrate an effect of lutein and elucidate the associated mechanisms underlying it, our study has some limitations. First, all experiments were performed in an in vitro hepatocellular model using FFA-treated HepG2 cells, which may not fully recapitulate the complexity of MASLD in vivo, including immune modulation, adipokine signaling, and gut-liver interactions. Second, while the chloroquine and rapamycin data support autophagy-dependency, additional mechanistic studies are needed to define the precise molecular steps by which lutein induces TFEB nuclear translocation and whether this involves mTOR-dependent or mTOR-independent signaling. Third, while we provide a functional readout of lipids in the extracellular medium as evidence of lipid efflux, dedicated assays specifically quantifying lysosomal exocytosis would further strengthen these findings. In addition, no quantitative assay of cell viability or membrane integrity was performed. Although routine qualitative examination of the cultures by phase-contrast microscopy did not reveal evident cell detachment or a reduction in cell density across treatment groups, and comparable cell morphology can also be appreciated in the representative immunofluorescence images presented throughout the manuscript, we cannot exclude subtle membrane damage or low levels of cell death as contributors to the extracellular triglyceride measurements. Finally, lutein was administered simultaneously with FFAs; therefore, our experimental design primarily evaluates its ability to prevent lipid droplet accumulation rather than to reverse pre-existing steatosis. This preventive approach is nevertheless physiologically relevant, as lutein is a dietary carotenoid that can be consumed chronically as part of a healthy diet. Accordingly, our findings support the potential of lutein as a nutritional strategy to reduce the development of hepatic steatosis. Whether lutein can also promote the resolution of established steatosis through TFEB-mediated autophagy remains to be determined and should be addressed in future in vivo studies. Despite these limitations, the convergence of multiple readouts, TFEB localization, LC3-LD colocalization, LD morphology, and extracellular triglycerides strengthens the plausibility of a coordinated cellular response to lutein.

From a phytopharmacological perspective, these findings highlight lutein as a naturally derived antioxidant that modulates hepatocellular lipid handling through TFEB- and autophagy-related pathways, with potential implications for MASLD.

Future studies should evaluate the impact of lutein on TFEB-mediated lipid clearance in animal models of steatosis, as well as its safety and efficacy in combination with existing lifestyle or pharmacological-based interventions. In summary, lutein emerges as a candidate natural antioxidant compound for further investigation as a supportive strategy in the context of metabolic-associated liver disease, acting through TFEB-regulated mechanisms of lipid clearance.

## 5. Conclusions

Overall, our data suggest that lutein modulates lipid homeostasis in FFA-treated HepG2 cells, an effect associated with TFEB nuclear translocation, autophagy-related responses, and changes in lysosomal organization. These findings are compatible with the involvement of autophagy–lysosomal pathways in the lipid-lowering effects of lutein, although additional studies will be required to establish the relative contribution of autophagosome formation, autophagic flux, and other mechanisms, including extracellular lipid release and lysosomal exocytosis.

## Data Availability

All data necessary to support the findings of this study are included in the article and its Supplementary Information. Additional data, if required, are available from the corresponding author upon reasonable request.

## Supplementary Materials

The following supporting information can be downloaded at: https://www.mdpi.com/article/10.3390/antiox15081042/s1, Figure S1. LAMP1 distribution and intensity in FFA-treated HepG2 cells; Figure S2. Effect of lutein on reactive oxygen species and TFEB-GFP nuclear translocation; Figure S3. LC3-I/LC3-II and p62 Western blot (n = 1); Figure S4. TFEB silencing and its effect on extracellular triglycerides and β-hexosaminidase release (n = 1); Table S1. Primer sequences (from 5′ to 3′).
