# Supplementary material for: Lutein Attenuates Lipid Accumulation in Association with TFEB Nuclear Translocation and Autophagy–Lysosomal Responses in a Cellular Model of Hepatic Steatosis
Source: Antioxidants (Basel). 2026 Aug 21;15(8):1042. doi: 10.3390/antiox15081042 (PMC13509890; doi:10.3390/antiox15081042)
Supplement: Supplementary file 1 [file antioxidants-15-01042-s001.zip › Supplementary Figure S2.pdf]

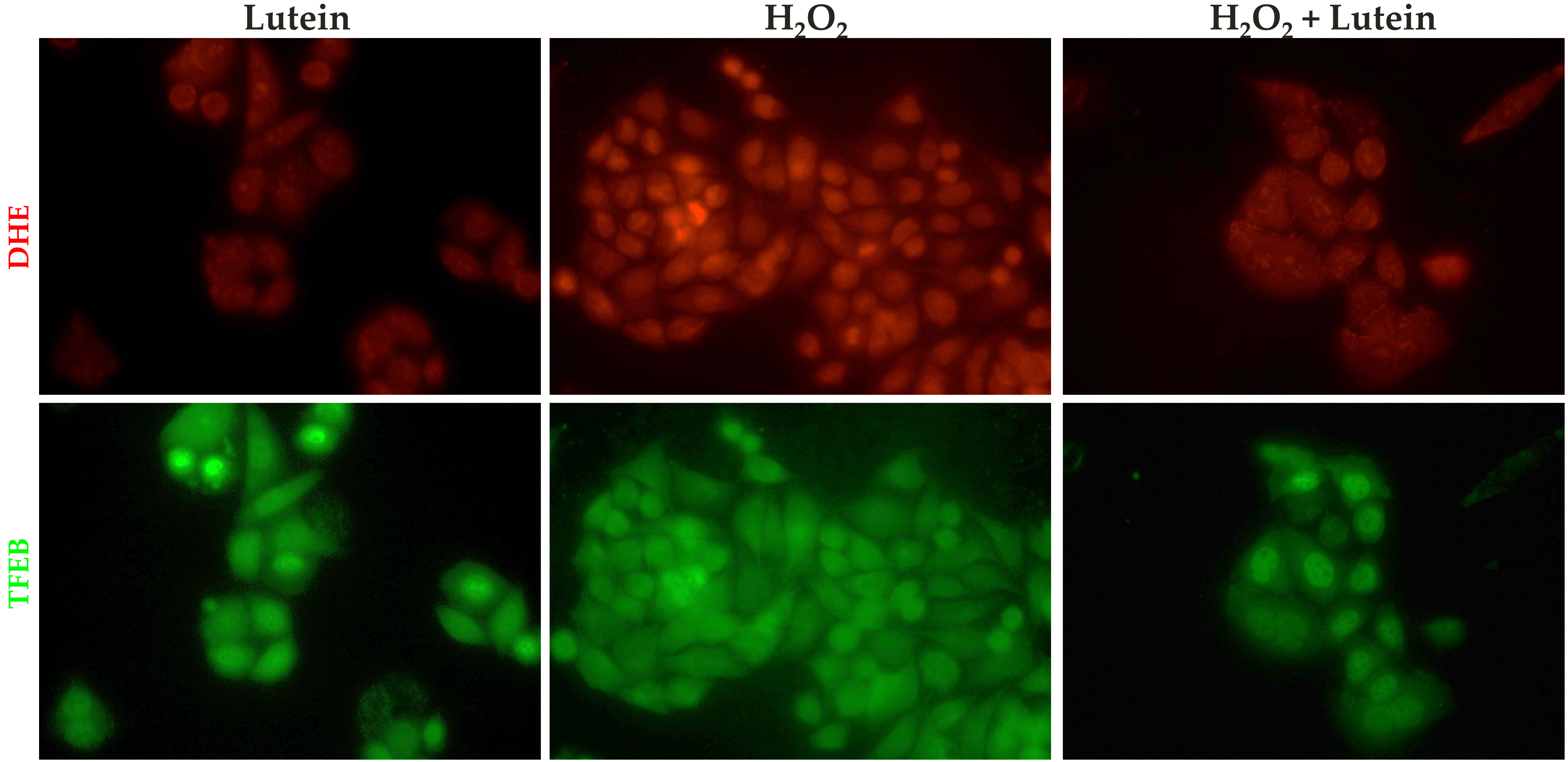
Lutein
H_2O_2
H_2O_2 + Lutein
DHE
TFEB
