# Supplementary material for: Lutein Attenuates Lipid Accumulation in Association with TFEB Nuclear Translocation and Autophagy–Lysosomal Responses in a Cellular Model of Hepatic Steatosis
Source: Antioxidants (Basel). 2026 Aug 21;15(8):1042. doi: 10.3390/antiox15081042 (PMC13509890; doi:10.3390/antiox15081042)
Supplement: Supplementary file 1 [file antioxidants-15-01042-s001.zip › Supplementary Figure S3.pdf]

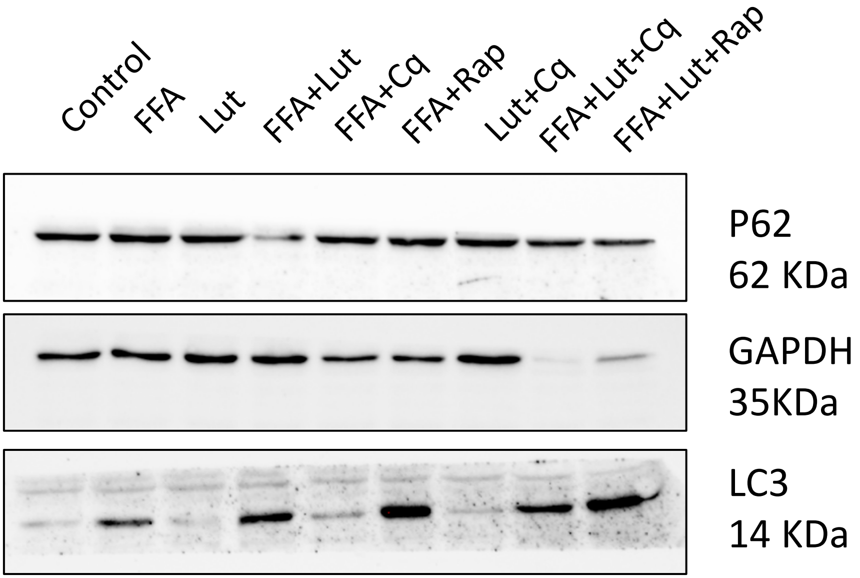
Control
FFA
Lut
FFA+Lut
FFA+Cq
FFA+Rap
Lut+Cq
FFA+Lut+Cq
FFA+Lut+Rap
P62
62 KDa
GAPDH
35KDa
LC3
14 KDa
