# Supplementary material for: Lutein Attenuates Lipid Accumulation in Association with TFEB Nuclear Translocation and Autophagy–Lysosomal Responses in a Cellular Model of Hepatic Steatosis
Source: Antioxidants (Basel). 2026 Aug 21;15(8):1042. doi: 10.3390/antiox15081042 (PMC13509890; doi:10.3390/antiox15081042)
Supplement: Supplementary file 1 [file antioxidants-15-01042-s001.zip › Supplementary Figure S4.pdf]

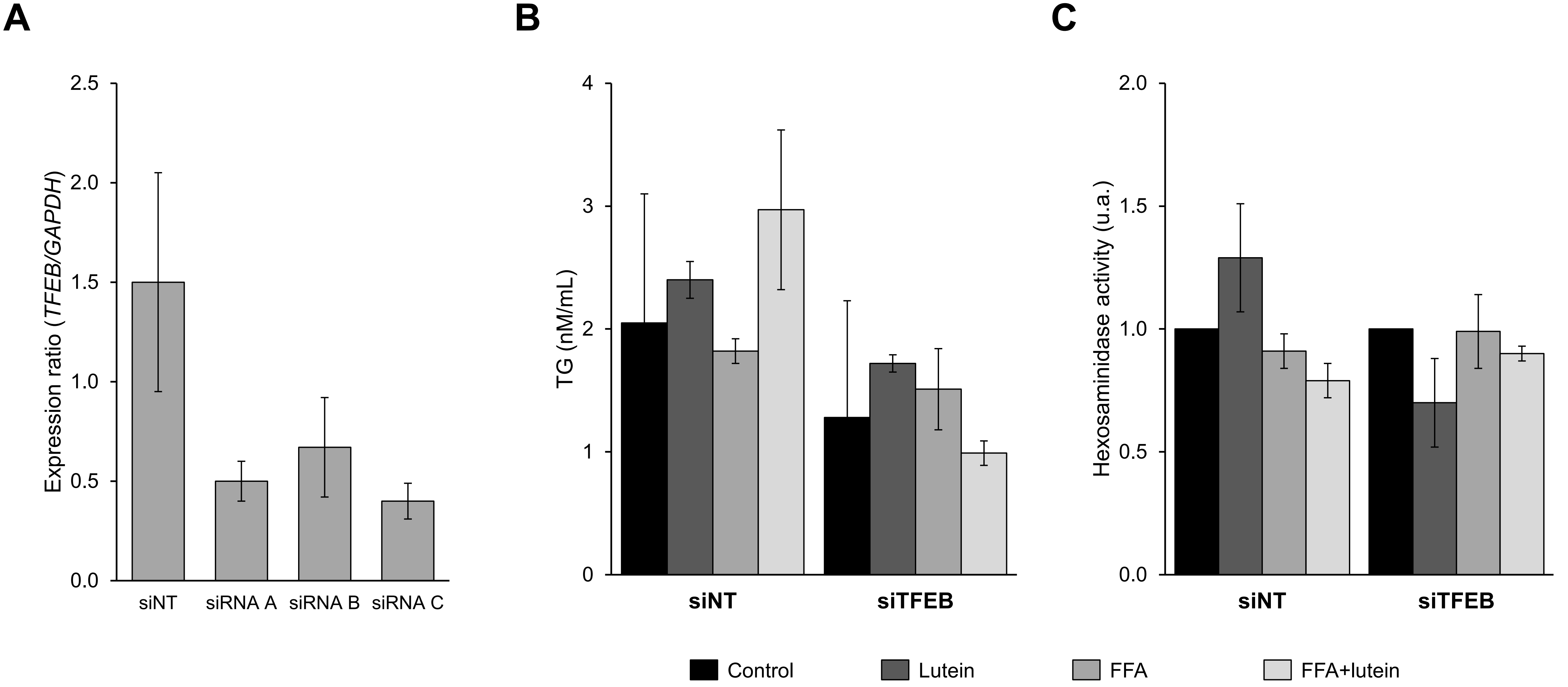

A
B
C
Expression ratio (TFEB/GAPDH)
2.5
2.0
1.5
1.0
0.5
0.0
siNT
siRNA A
siRNA B
siRNA C
TG (nM/mL)
4
3
2
1
0
siNT
siTFEB
Hexosaminidase activity (u.a.)
2.0
1.5
1.0
0.5
0.0
siNT
siTFEB
Control
Lutein
FFA
FFA+lutein
