# Supplementary material for: Lutein Attenuates Lipid Accumulation in Association with TFEB Nuclear Translocation and Autophagy–Lysosomal Responses in a Cellular Model of Hepatic Steatosis
Source: Antioxidants (Basel). 2026 Aug 21;15(8):1042. doi: 10.3390/antiox15081042 (PMC13509890; doi:10.3390/antiox15081042)
Supplement: Supplementary file 1 [file antioxidants-15-01042-s001.zip › Supplementary Table S1.pdf]

Supplementary Table S1. Primer sequences (from 5′ to 3′)

Gene	Forward (5′-3′)	Reverse (5′-3′)	Gene Accession Number
LAMP1	CCAACTTCTCTGCTGCCTTC	AGCAATCACGAGACTGGGG	NM_005561.4
SQSTM1	AAATGGGTCCACCAGGAAACTGC	TCAACTTCAATGCCCAGAGGGCT	NM_003900.5
MAP1LC3B	TGTCCGACTTATTCGAGAGCAGC	TTCACCAACAGGAAGAAGGCCTG	NM_022818.5
ATG9A	TTCCTTTGCCCTTATGGATG	AACCGCATCAAAGAAAGCTC	NM_024085.5
BECN1	GGTGTCTCTCGCAGATTCATC	TCAGTCTTCGGCTGAGGTTCT	NM_003766.5
TFEB	GCGGCAGAAGAAAGACAATC	ATTACATCCGGAGGATGCAG	NM_007162.3
GAPDH	ATGTTCGTCATGGGTGTGAA	AGGGGTGCTAAGCAGTTGGT	NM_002046.7
